# Exploring Medical Expenditure Clustering and the Determinants of High-Cost Populations from the Family Perspective: A Population-Based Retrospective Study from Rural China

**DOI:** 10.3390/ijerph15122673

**Published:** 2018-11-27

**Authors:** Shan Lu, Yan Zhang, Yadong Niu, Liang Zhang

**Affiliations:** 1 School of Medicine and Health Management, Tongji Medical College, Huazhong University of Science and Technology, Wuhan 430030, China; shanlu@hust.edu.cn (S.L.); nyadong@126.com (Y.N.); zhangliang@mails.tjmu.edu.cn (L.Z.); 2 Research Centre for Rural Health Service, Key Research Institute of Humanities & Social Sciences of Hubei Provincial Department of Education, Wuhan 430030, China

**Keywords:** medical expenditure, high-cost family, clustering, rural China

## Abstract

The costliest 5% of the population (identified as the “high-cost” population) accounts for 50% of healthcare spending. Understanding the high-cost population in rural China from the family perspective is essential for health insurers, governments, and families. Using the health insurance database, we tallied 202,482 families that generated medical expenditure in 2014. The Lorentz curve and the Gini coefficient were adopted to describe the medical expenditure clustering, and a logistic regression model was used to identify the determinants of high-cost families. Household medical expenditure showed an extremely uneven distribution, with a Gini coefficient of 0.76. High-cost families spent 54.0% of the total expenditure. The values for family size, average age, and distance from and arrival time to the county hospital of high-cost families were 4.05, 43.18 years, 29.67 km, and 45.09 min, respectively, which differed from the values of the remaining families (3.68, 42.46 years, 30.47 km, and 46.29 min, respectively). More high-cost families live in towns with low-capacity township hospitals and better traffic conditions than the remaining families (28.98% vs. 12.99%, and 71.19% vs. 69.6%, respectively). The logistic regression model indicated that family size, average age, children, time to county hospital, capacity of township hospital, traffic conditions, economic status, healthcare utilizations, and the utilization level were associated with high household medical expenditure. Primary care and health insurance policy should be improved to guide the behaviors of rural residents, reduce their economic burden, and minimize healthcare spending.

## 1. Introduction

Medical expenditure clustering is a phenomenon that reflects the uneven distribution of medical expenditure among a given population [1]. In recent years, medical expenditure clustering has been discussed intensively in healthcare research because a frequently cited report showed that the costliest 5% of patients accounted for approximately 50% of the total annual health spending [2]. A large number of studies within the United States have confirmed that a small group of the most expensive patients, identified as the “high-cost” (HC) patients, spent a disproportionate amount of healthcare expenditure [3,4,5]. A few studies from European countries and Taiwan with different health insurance schemes and healthcare delivery systems showed similar findings [5,6,7]. HC patients attract increasing attention not only because they are costly, but also because they use more healthcare resources, with sometimes unavoidable inappropriate utilization [8]. Governments, health insurers, and providers are increasingly focusing on HC patients and attempting to improve means of delivering care for them to enhance health outcomes and reduce spending [9,10].

Previous studies on health expenditure clustering used the individual as a unit in analysis. Only a few investigations have focused on this problem at the household level. We found only one study conducted from the family perspective about the HC population in China [11]. The study investigated 12,600 families in Jiangsu Province, an economically developed urban region in China, and used household out-of-pocket medical expenditure for analysis. Results showed that HC families accounted for 44.9% of the total expenditure and thus represented a heavy health burden. Identifying the clustering of household health expenditure and the characteristics of HC families is essential, especially in poor rural China. Three reasons for such needs are as follows.

First, families are the most basic and important social units in which illness occurs, lingers, or resolves [12,13], and families are the decisive factors in their members’ healthcare utilization and expenditure payments [11]. Second, family members live together within a common home environment and share the same lifestyle and even beliefs relating to illness and health, thereby influencing one another’s health-seeking behaviors [12,14]. Empirical evidence confirms that family members have similar healthcare utilization [12,14,15,16,17]. The average family size in rural China is larger than that of urban families [18], given that many rural families comprise three or four generations living under one roof. The presence of special family members (e.g., children, the elderly, or patients with chronic diseases) may cause the whole family to use more healthcare resources [11,14], with even unnecessary utilization, thus resulting in increased expenditure. Third, identifying the characteristics of HC families will attract the particular interest of Chinese health insurance policymakers. In the past few decades, the Chinese government has established three basic medical insurance schemes to alleviate the economic burden of residents, namely, the Urban Employee Basic Medical Insurance scheme (since 1998), the Urban Resident Basic Medical Insurance scheme for urban residents (since 2007) [19], and the New Rural Cooperative Medical Scheme (NCMS) for rural residents (since 2003) [20]. The NCMS, unlike the two other schemes for urban residents, considers the family as a unit to cover rural residents. Although the NCMS has covered more than 98% of the rural families since 2012, it faces considerable challenges due to a glaring defect [21,22]. Given that the medical expenditure is paid jointly by the NCMS and patients’ out-of-pocket payments and NCMS funding is managed at the household level, reducing the health expenditure of HC families will lighten the burden of the health insurance fund and of the HC families.

Given the above findings, identifying the HC families in rural China is an essential issue. This study aims to analyze the clustering of household expenditure using the NCMS database, to explore the characteristics of HC families, and to ascertain the determinants of the annual household health expenditure in general. We use gross household medical expenditure rather than out-of-pocket expenditure for analysis because the former is more significant than the latter to the government and health insurers. The results of this study can shed light on improved healthcare delivery and future health insurance policymaking in rural China.

## 2. Materials and Methods

### 2.1. Study Setting

This household perspective retrospective study was performed in Macheng City, Hubei Province. Macheng is a county-level rural area in Central China that has 889,160 rural residents, 255,151 rural families, and a GDP per capita of 3706.5 United States dollars (US$) (Exchange rate in 2014: RMB¥6.14 to US$1.00). All rural families in Macheng are covered by the NCMS [23], which offers reimbursement for outpatient and inpatient services in medical institutions at different levels, including village clinics, township hospitals, county hospitals, hospitals outside the county, and some private medical institutions. Macheng has 207 village clinics, 22 township hospitals (one township hospital in each town), and 2 county hospitals. The medical care utilization and medical expenditure of every family member covered by the NCMS are recorded in the NCMS database.

In 2014, the NCMS only reimbursed outpatient expenditure for primary health care institutions (including village clinics and township hospitals), and the NCMS reimbursement rate (the proportion of expenditures the insurer reimburses until the cap is reached) for primary care institutions was 50%. The annual outpatient reimbursement cap, the maximum amount that NCMS can reimburse per insured family, was US$58.6. For inpatient expenditure, the reimbursement rates for township hospitals, the first county hospital, and the second county hospital were 90%, 85%, and 75% respectively. The annual inpatient reimbursement cap was US$16,286.6.

### 2.2. Data Sources

This study targeted rural families in which at least one member had used medical care. The NCMS database includes only family members who have utilized medical care. Thus, to collect all information needed for the target families, we integrated the 2014 NCMS database of Macheng and the NCMS register, which includes all family members covered by NCMS, for analysis. Using the integrated database, we calculated the annual household medical expenditure (including gross, outpatient, and inpatient expenditures), frequency of medical care utilization (including outpatient visits to and hospitalizations in hospitals at different levels), and other household characteristics (including family size, average age, presence of the elderly over 60 years old, and presence of children under 6 years old) of each family on the basis of their NCMS family ID. According to the household annual medical expenditure, the target families were sorted in descending order. The top 5% of the households were defined as HC families. 

Besides the annual medical expenditure, the frequency of medical care utilization, and the basic sociological characteristics of each household, several variables that may influence household medical expenditure were designed in this research. We classified the township hospitals in Macheng into three categories (low, medium, or high level) according to their resource-based and service capacities. For each town, the arrival time to and distances from the county hospital, the landform (plain, hilly, hilly and mountainous, or mountainous), and the traffic condition (national standard roads, provincial standard roads, or county roads) were determined using Google Maps. In addition, we described the families as low income households or non-low income households to represent their economic status according to the list of low-income households in the Poverty Alleviation Information System. 

### 2.3. Statistical Analysis

The Lorentz curve and the Gini coefficient were employed to describe and measure the clustering of household medical expenditure, respectively. Lorentz curve is a graphical representation of the distribution of household medical expenditure, and Gini coefficient is a common measure of distribution derived from the Lorentz curve. The Gini coefficient ranges from 0 to 1, with 0 indicating absolutely even distribution of medical expenditure, and 1 indicating perfectly uneven distribution. Therefore, a larger Gini coefficient represents a higher degree of household medical expenditure clustering. Analysis of variance and χ^2^ tests were used to compare the characteristics of the HC and the remaining (RM) families in terms of medical expenditure, medical care utilization, family characteristics, capability of township hospital, landform, traffic condition, and economic status in SPSS 22.0 (SPSS Inc., Chicago, IL, USA; http://www.spss.com). The determinants of high household medical expenditure of HC families were identified through a logistic regression model in SPSS 22.0. The independent variables included family size, average age, having at least one member older than 60, having at least one member younger than 6, economic status, distance from and arrival time to the county hospital, capacity of township hospital, landform, traffic condition, number of outpatient visits and admissions to medical institution at different levels. Definitions of all variables included in the logistic regression model were shown in Appendix A.

### 2.4. Ethics Approval

The study protocol conformed to the guidelines of the Ethics Committee of the Tongji Medical College of Huazhong University of Science and Technology and was registered in the Chinese Clinical Trial Registry (ChiCTR-OOR-14005563).

## 3. Results

Household medical expenditure in this study refers to the sum of outpatient and inpatient expenditures (including drug costs). Among the 255,151 families covered by NCMS, 202,482 families utilized outpatient or inpatient services and generated medical expenditure in 2014. The total population in these families was 748,334, of whom 478,051 had used outpatient or inpatient services.

### 3.1. Clustering of Household Medical Expenditure

Annual household medical expenditure was ranked from highest to lowest, and the total medical expenditure and cumulative medical expenditure of different household ranking intervals were calculated. Table 1 shows the clustering of the rural household medical expenditure of Macheng in 2014. The top 20% of families spend 83.5% of the total expenditure. The costliest HC families account for only 5% of the total families and spend 54.0% of the total expenditure.

**Figure 1:**
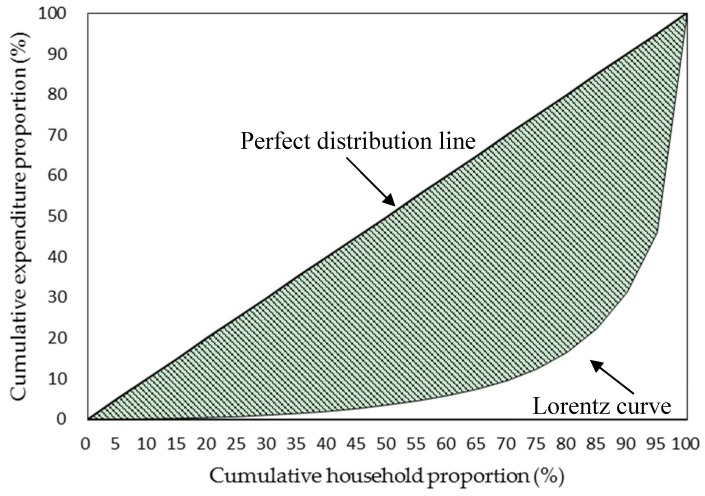
Lorentz curve of household medical expenditure clustering in Macheng in 2014.

Figure 1 shows that the Lorentz curve depicting household medical expenditure per share of household was far from the perfect distribution line. This represents that the medical expenditures were uneven distributed among rural households. The Gini coefficient derived from the Lorentz curve is 0.76, which is close to 1, indicating high level of clustering.

### 3.2. Medical Expenditure

The proportions of HC and RM families that generated outpatient expenditure in 2014 exceed 90%, and the value is slightly higher for the RM families (98.42%) than the HC families (93.5%) (Table 2). The proportions of families with inpatient expenditure differ significantly between the two groups (100% for the HC families, 25.58% for the RM families). As for the expenditure composition of the two groups, outpatient expenditure accounts for only 0.1% of the total household expenditure for HC families, whereas their inpatient expenditure accounts for 99.9%. Conversely, for the RM families, outpatient expenditure represents much higher share (75.75%). We also calculated the average household medical expenditure for the HC families (US$5078.9) and the RM families (US$227.5). The HC group is costlier than the RM group in inpatient expenditure (US$ 4832.0 vs. US$140.8) and outpatient expenditure (US$46.9 vs. US$86.7).

### 3.3. Medical Care Utilization

Table 3 lists the outpatient and inpatient service utilization of the HC and RM families in 2014. HC families utilize more services regardless of the service type (outpatient and inpatient services) or the service levels, and all the differences are statistically significant (*p* < 0.001). HC families use slightly more outpatient services (22.47 ± 22.99 annual outpatient visits) than the RM families (17.06 ± 18.48). As expected, the HC families are far more inclined to utilize inpatient services (3.83 ± 6.46 average annual hospitalization) than the RM families (0.37 ± 0.97 average annual hospitalization). Regarding inpatient levels, the disparity between the two groups is greatest for the “outside the county” level, with the annual hospitalization of HC families (0.99 ± 2.23) being 22 times that of the RM families (0.045 ± 0.25).

### 3.4. Household Characteristics

Table 4 shows the household characteristics for HC and RM families. Specifically, the HC families have larger family sizes (4.05 ± 1.63) than RM families (3.68 ± 1.51). HC families are also older (43.18 ± 11.97) than the RM families (42.46 ± 12.76). The proportion of families with at least one family member older than 60 years old for HC families is 49.47%, which is more than that of the RM families (42.19%). Similarly, the HC group has more families with at least one child under 6 (12.44%) than the RM families (9.29%). Both distance from and arrival time to the county hospital for HC families (29.67 ± 15.58, 45.09 ± 18.52) are shorter than those of the RM families (30.47 ± 15.21, 46.29 ± 18.27). As for the capacity of township hospitals, more HC families reside in towns with low-capacity township hospitals (28.98%) than RM families (12.99%), and less of the former live in towns with high-capacity township hospitals (15.46%) than the latter (30.05%). In addition, RM families are more likely to live in regions with poor-quality roads (11.99% and 30.40% mountainous and county roads, respectively) than the HC families (11.64% and 28.81%, respectively). Moreover, more low income households are found in the HC group (11.59%) than in the RM group (9.80%). All differences are statistically significant (*p* < 0.05).

### 3.5. Determinants of High Medical Expenditure for High-Cost Families

Determinants of high medical expenditure for HC families were examined using a logistic regression model (Table 5). The regression model used a stepwise selection method. Results of the logistic regression model (with an variable the elderly excluded in the final model, *p* = 0.26) show that large families, old average age, lack of child, short arrival time to the county hospital, and low incomes are likely to characterize HC families. Moreover, families located in regions with low township hospital capacity and good road conditions (national roads/provincial roads) are at higher risk of being HC families. Families living in hilly regions and hilly and mountainous regions are more likely to be HC families than are those residing in mountainous regions. As for outpatient and inpatient service utilization of hospitals at different levels, the frequency of inpatient admission in hospitals outside the county (the highest level) has the highest value of odds ratio (OR) (14.887).

## 4. Discussion

### 4.1. Clustering of Most Rural Medical Expenditure within Few Costly Families

The Lorentz curve and the Gini coefficient (0.76) described in this study demonstrated an extremely uneven distribution of the annual medical expenditures of the rural families. The top 20% costliest families consumed 83.5% of the medical expenditure, which perfectly accorded with the famous Pareto principle (80/20 rule). The costliest 5% of the rural families accounted for 54% of the total expenditure. This finding was consistent with research on this issue at the individual [3,4] and family levels [11]. Similar to the medical expenditure clustering between families, the expenditure within an HC family also presented an extremely uneven distribution between outpatient and inpatient services. Although HC families were inclined to use both outpatient and inpatient services, nearly all of their expenditure (99.9%) came from hospitalization rather than outpatient visits, and this finding was in line with those of Driessen et al. and Fang et al. [24,25]. 

The average total expenditure of HC families was US$5078.9, which was nearly 22 times that of RM families (US$227.5). Although the NCMS has achieved wide coverage, its effective reimbursement rate is still relatively low [26]. For HC families, only approximately 50% of the total expenditure can be reimbursed by the NCMS, which means that about US$2539.4 on average should be borne by HC families themselves. Accordingly, for rural residents with a disposable income per capita less than US$1628.7 [27], the medical expenditure could be catastrophic for HC families (where the average disposable income per family is approximately US$6026.1 with a 3.7 rural family size). This outcome supported the conclusion of Miao et al. [11]. Therefore, the NCMS provides limited protection to HC families, who need more attention than do others. At the same time, the high cost of HC families is not always reasonable. Results of this study showed that the rural HC families were more inclined to utilize inpatient services, especially services provided by the county hospital (annual hospitalizations 2.36 ± 6.18). According to the studies of Yan et al. [28] and Xiaoyan et al. [29], the inappropriate admission rate for a county hospital in a county of China reached as high as 12.14%, and that for township hospitals was approximately 20%. 

According to this analysis, the few HC families should be the focus of the government and of NCMS not only because doing so may help the government and health insurers achieve more with less, as proposed by the Pareto principle, but also because HC families are disproportionately likely to suffer from catastrophic health expenditure and can enjoy reduced costs by simultaneously avoiding inappropriate utilization at the same time.

### 4.2. Impacts of Family Characteristics on Family Medical Expenditure

As for the demographic characteristics, the logistic regression model showed that family size and average age of a family had a significant positive effect on annual medical expenditure, and this result was consistent with that of the study of Fang et al. on gross medical expenditure in Taiwan [25]. Remarkably, families with children under 6 years old were less likely to be HC families. According to a study by Sefehri [14], the presence of children under 6 years old in a family can positively increase the health-seeking behavior of adult family members. Given Sefehri’s conclusion and the result of this study, we could infer that families with children under 6 years old may utilize more medical services but do not spend much because the entire family may be relatively younger (average age 32.15 ± 5.96, families without children: 43.58 ± 12.73), healthier, and would use more outpatient rather than inpatient services. In addition, families with the elderly over 60 years old were not taken as potential predictors for high expenditure, which seemed to be in contrary to many previous studies [7,30]. However, upon further consideration, we could explain that the key determinant of HC families was not whether the family had old members but whether the old members had poor health status and increased healthcare utilization.

Distance from and arrival time to the county hospital were significant indicators for HC families, with the distance as positively significant and time as negatively significant. A comparison of Table 4 and Table 5 reveals contradictory results for distance. Thus, we could not draw a definite conclusion about the impact of distance on household expenditure. However, families with shorter arrival time to the county hospital were likely to be HC families. In general, every county in China has one county hospital that provides the best medical services within the county; naturally, these hospitals are also the most expensive in each county. Yan-Ning Li et al. [31] found that travel time to the nearest healthcare institution was a determinant of health service utilization. Understandably, rural residents seek better and even more services if doing so will not consume much time. Besides, families living in regions with the worst traffic condition (county roads) were least likely to be HC families possible due to their limited geographical access to expensive services.

### 4.3. Increase in Capacity of Primary Care 

Results of the logistic regression model indicated that the OR of different categories of the capacity, outpatient utilization, and inpatient admission at different levels revealed two severe phenomena in the Chinese healthcare system. First, the capacity of primary care (including services provided by village clinics and township hospitals) cannot meet the health needs of residents; second, the residents’ preference for hospitals at high levels leads to “*kan bing nan, kan bing gui*” (“Medical treatment is difficult to access and expensive”) [32]. In predicting an HC family, families living in towns with medium- or low-capacity township hospitals had odds of 1.679 or 3.152 times higher the odds of those with high-capacity township hospitals, respectively. Thus, if the township hospitals are incompetent and the primary care they provide cannot satisfy the population, then rural residents may seek health services from county hospitals or even hospitals outside the county, which can definitely be more expensive. In fact, the present primary care is inadequate in terms of both resources and services. On the one hand, studies have shown that the Chinese primary healthcare system lacks qualified workforce, high-value medication, and integrated health information technology systems [33,34,35,36]; on the other hand, the quality of care provided by the primary care system is poorly characterized by a high proportion of antibiotic and injection use and low patient satisfaction [35,37,38]. The insufficient capacity of primary healthcare and the residents’ freedom to choose first-contact healthcare institutions drive patients to high-level hospitals. 

Moreover, this study confirmed that the outpatient service utilization from institutions at different levels (clinics, township hospital, and county hospital) had a rising trend of ORs at 1.003, 1.005, and 1.062, respectively, and so did inpatient service utilization (township hospital, county hospital, and hospital outside the county with ORs of 1.943, 3.418, and 14.887, respectively). Clearly, the higher the level of medical institutions visited, the higher the possibility of the patients becoming HC families. A survey on 784 rural residents found that the rate of inappropriate choice of high-level hospitals was 50.10% [39]. In other words, approximately half the residents should have been treated in the primary care system rather than in high-level hospitals for common medical needs according to the government’s positioning for institutions at different levels. If such population could be served in the primary care system, then health expenditure would be reduced substantially.

### 4.4. Limited Impacts of Health Insurance on Shaping Health-Seeking Behavior and on Rural Residents’ Financial Risk Protection

The NCMS provides limited coverage for outpatient or primary care provided by clinics or township hospitals. Households are the basic unit for rural residents in joining the NCMS. This scheme sets a family account for each insured family to be used in reimbursing their outpatient expenditure (for primary care only), and all the family members can share the balance in the family account. However, a research has shown that the low reimbursement cap encourage rural residents to seek healthcare from high-level hospitals after the annual cap is reached [35]. For inpatient services, patients are more willing to obtain services in high-level hospitals rather than in primary care facilities because the disparities of the reimbursement rates between township hospitals and secondary/tertiary hospitals are small [35,40]. One study revealed that an increase in outpatient expenses of US$1 could result in a reduction in inpatient expenses by US$6 [41]. If the NCMS policies favored primary and outpatient care and could correct the health-seeking behavior of rural residents, then cost saving would be easier. However, such is not the case at present. 

In addition to shaping health-seeking behavior of residents, the NCMS policies need to be improved to better protect HC families from financial risk. Though the high cost of HC families is not always reasonable, there are still quite a little families with serious health problem and reasonably generate high medical expenditure. Considering that these families are vulnerable to catastrophic health expenditure, the NCMS needs to pay special attention to them. Given that HC population is characterized by chronic disease [4,5] and the NCMS’s coverage of pharmaceuticals and outpatient services is limited [30], household members with chronic disease, especially those from low income families, may refrain from seeking care until advanced illness occurs. Therefore, to relieve the disease burden of HC families, the NCMS could extend insurance coverage to long-term care for patients with chronic disease; In addition, the reimbursement cap and rate need to be increased for specific diagnoses which are closely linked with high cost. 

It is seems an universal phenomenon that a vital few population (or families) accounts for a large proportion of the total expenditure, no matter what health insurance scheme or health delivery system and how the economic developed. It is true for the United States, with a its commercial health insurance system, for several European countries [5,6], and China (the mainland) with statutory health insurance system, and even for Taiwan, which has a similar national health service system to that of the United Kingdom [7]. For economically developed countries, health projects or plans could be implemented to reduce unreasonable and avoidable health service utilization, since some actions have already turned out valuable in the United States [8,10]. For economic developing countries with relatively weaker primary health care system and scarce resources, improving the health delivery system and the health insurance scheme should also be a priority. 

Though the unit of analysis was a family rather than an individual, it would not be subject to an ecological fallacy for at least two reasons: first, lots of studies verified that family members influence one another’s health-seeking behaviors and have similar healthcare utilization [12,14,15,16,17], especially in rural China where a household is a unit for health insurance coverage. Therefore, conclusions of this study for high-cost families apply to individuals. For example, all family members probably seek health service at the secondary or tertiary hospitals rather than primary care institutions once they reach their family’ s reimbursement cap. If a household is close to the county hospital, all family members very likely go there rather than township hospitals and use expensive services, thus increasing the likelihood of becoming a high-cost family. Second, because household is an insured unit for the NCMS, finding out the determinants of high-cost families rather than individuals may be more crucial to the NCMS.

### 4.5. Strengths and Limitations

The strength of this study lies in expanding the scope of past research on the HC population to include the clustering of medical expenditure among the whole population, from focusing on individuals to families, and from evaluating economically-developed areas to economically-developing rural areas. However, this work has two limitations. First, the economic status included in this study involves a categorical variable (low income or non-low income family). Such status is not as precise as using continuous data on household income. Second, in considering the determinants of household medical expenditure, data on the health status of family members are lacking. Further research on the HC population from the family perspective should include additional information about the economic and health statuses of families for analysis.

## 5. Conclusions

Household medical expenditure is unevenly distributed in rural China; the vital few (costly families) account for a large proportion of the total expenditure. Families living in towns with low-capacity township hospitals and good traffic conditions are likely to be HC families. We suggest improvements in the services provided by the primary care system. Moreover, NCMS policies should favor primary and outpatient care to correct the health-seeking behavior and reduce the unnecessary healthcare utilization of rural residents. These actions will alleviate the economic burden of rural families and reduce healthcare spending.

## Figures and Tables

**Table 1:** Rural household medical expenditure clustering in Macheng in 2014.

Household Ranking (%) ^1^	Total Medical Expenditure (US$10 Thousand)	Cumulative Medical Expenditure (US$10Thousand)	Cumulative Percentage of Medical Expenditure (%)
0–5	5141.9	5141.9	54.0
5–10	1394.2	6536.1	68.7
10–20	1413.1	7949.2	83.5
20–30	662.8	8612.0	90.5
30–40	358.3	8970.3	94.2
40–50	219.3	9189.6	96.5
50–60	143.2	9332.8	98.1
60–70	92.3	9425.0	99.0
70–80	55.4	9480.4	99.6
80–90	28.4	9508.8	99.9
90–100	9.3	9518.1	100.0

^1^ Household ranking interval does not include the left end point.

**Table 2:** Medical expenditure of different family groups in Macheng in 2014.

Medical Expenditure	HC Families *n* = 10,124	RM Families *n* = 192,358
Proportion of families with outpatient expenditure (%)	93.5	98.4
Proportion of families with inpatient expenditure (%)	100.0	25.6
Ratio of outpatient expenditure to total expenditure (%)	0.1	75.8
Ratio of inpatient expenditure to total expenditure (%)	99.9	24.3
Average outpatient expenditure in US$ (x ± s ^1^)	246.9 ± 517.8	86.7 ± 103.1
Average inpatient expenditure in US$ (x ± s)	4832.0 ± 5025.8	140.8 ± 319.4
Average total expenditure in US$ (x ± s)	5078.9 ± 5015.5	227.5 ± 349.9

^1^ x ± s: mean ± standard deviation; HC: high-cost; RM: remaining.

**Table 3:** Medical care utilization of different family groups in Macheng in 2014.

Medical Care Utilization	HC Families *n* = 10,124	RM Families *n* = 192,358	*p* Value
Annual outpatient visits per household (x ± s)	22.47 ± 22.99	17.06 ± 18.48	<0.001
Outpatient level			
Village clinic (x ± s)	12.80 ± 18.95	11.34 ± 16.56	<0.001
Township hospital (x ± s)	6.45 ± 12.96	4.52 ± 9.48	<0.001
County hospital (x ± s)	3.21 ± 4.61	1.20 ± 2.64	<0.001
Annual hospitalizations per household (x ± s)	3.83 ± 6.46	0.37 ± 0.97	<0.001
Inpatient level			
Township hospital (x ± s)	0.48 ± 1.33	0.11 ± 0.43	<0.001
County hospital (x ± s)	2.36 ± 6.18	0.21 ± 0.81	<0.001
Outside the county (x ± s)	0.99 ± 2.23	0.045 ± 0.25	<0.001

**Table 4:** Household characteristics of different family groups in Macheng in 2014.

Household Characteristics	HC Families *n* = 10,124	RM Families *n* = 192,358	*p* Value
Family size (x ± s)	4.05 ± 1.63	3.68 ± 1.51	<0.001 *
Average age within family ^1^ (x ± s, y)	43.18 ± 11.97	42.46 ± 12.76	<0.001 *
Family with the elderly ^1^ (*n*, %)			
Yes	4567 (49.47)	74,042 (42.19)	<0.001 ^†^
No	4665 (50.53)	101,472 (57.81)	
Family with children ^1^ (*n*, %)			
Yes	1148 (12.44)	16,301 (9.29)	<0.001 ^†^
No	8084 (87.56)	159,213 (90.71)	
Distance from county hospital (x ± s, km)	29.67 ± 15.58	30.47 ± 15.21	<0.001 *
Arrival time to county hospital (x ± s, min)	45.09 ± 18.52	46.29 ± 18.27	<0.001 *
Capacity of township hospital (*n*, %)			
Low	2934 (28.98)	24,991 (12.99)	<0.001 ^†^
Medium	5625 (55.56)	109,571 (56.96)	
High	1565 (15.46)	57,796 (30.05)	
Landform (*n*, %)			
Plain	3952 (39.04)	75,408 (39.20)	0.007 ^†^
Hilly	2418 (23.88)	47,499 (24.69)	
Hilly and mountainous	2576 (25.44)	46,396 (24.12)	
Mountainous	1178 (11.64)	23,055 (11.99)	
Traffic condition (*n*, %)			
National standard roads	4346 (42.93)	86,033 (44.73)	<0.001 ^†^
Provincial standard roads	2861 (28.26)	47,850 (24.88)	
County roads	2917 (28.81)	58,475 (30.40)	
Low income family ^2^ (*n*, %)			
Yes	1173 (11.59)	18,830 (9.80)	<0.001 ^†^
No	8944 (88.41)	173,257 (90.20)	

^1^ Given missing data (8.76% of the 202,482 families with at least one member lack age data), 184,746 families were included in the analysis; ^2^ Given missing data (278 families out of the 202,482 families lack economic status data), 202,204 families were included in the analysis; * *t*-test; ^†^ Pearson’s χ^2^ test.

**Table 5:** Factors associated with annual household expenditure of HC families (*n* = 184,013).

Covariate	Wals	*p* Value	OR	95%CI for OR	
				Lower	Upper
Family size	14.929	<0.001	1.047	1.023	1.072
Average age	12.801	<0.001	1.005	1.002	1.008
Child^1^	42.475	<0.001	0.722	0.654	0.796
Distance	3.940	0.047	1.005	1.000	1.009
Time	31.657	<0.001	0.990	0.986	0.993
Capacity ^1^	394.599	<0.001			
Low	390.004	<0.001	3.152	2.813	3.533
Medium	94.979	<0.001	1.679	1.513	1.864
Landform ^1^	578.957	<0.001			
Plains	53.285	<0.001	0.601	0.524	0.689
Hilly	11.002	0.001	1.278	1.106	1.478
Hilly and mountainous	132.970	<0.001	2.147	1.886	2.445
Traffic ^1^	51.853	<0.001			
National roads	19.433	<0.001	1.223	1.119	1.338
Provincial roads	51.729	<0.001	1.461	1.317	1.620
Low-income family ^1^	7.108	0.008	1.129	1.033	1.235
Outpatient_clinic	12.502	<0.001	1.003	1.001	1.005
Outpatient_township	13.518	<0.001	1.005	1.002	1.007
Outpatient_county	274.532	<0.001	1.062	1.055	1.070
Inpatient_township	1865.052	<0.001	1.943	1.885	2.002
Inpatient_county	8532.354	<0.001	3.418	3.330	3.508
Inpatient_outside	10,031.670	<0.001	14.887	14.121	15.695
Constant	2376.769	<0.001	0.004		
−2Log likelihood	39,848.179				
Nagelkerke R^2^	0.504				
Percent correctly predicted	96.4%				

^1^ Reference category: The elderly (families with no elderly), child (families with no child), Capacity (families located in regions with high-capacity township hospital), landform (families in mountainous regions), traffic (families located in regions with county roads), and low-income family (non-low income families).

## Appendix A

**Table A1:** Variables list of logistic regression model.

Variables	Definition
Explained variable	
HC families	RM family = 0, HC family = 1.
Explanatory variable	
Family size	Number of family members in a household.
Average age	Average age of all family members
The elderly	Whether or not the family has at least one the elderly over 60 years old. Family without the elderly over 60 was a reference category.
Child	Whether or not the family has at least one child under 6 years old.Family without child under 6 was a reference category.
Distance	Distance between household and the county hospital
Time	Arrival time to the county hospital from the household by car
Capacity	Capacity of the township hospital in the town where the family was located, including three categories: high, medium and low. High-capacity township hospital was a reference category.
Landform	Landform of the town where the family was located, including four categories: mountainous, hilly and mountainous, hilly and plains. Mountainous was a reference category.
Traffic	Road condition of the town where the family was located, including three categories: national roads, provincial roads, and county roads. County road was a reference category.
Low income family	Whether or not the family is a low income family.Non-low income family was a reference category.
Outpatient_clinic	Number of outpatient visits to village clinics for each family in 2014
Outpatient_township	Number of outpatient visits to township hospitals for each family in 2014
Outpatient_county	Number of outpatient visits to county hospitals for each family in 2014
Inpatient_township	Number of admissions to township hospitals for each family in 2014
Inpatient_county	Number of admissions to county hospitals for each family in 2014
Inpatient_outside	Number of admissions to hospitals outside the county for each family in 2014
